# Hepatocellular Carcinoma in Malaysia and Its Changing Trend

**DOI:** 10.5005/jp-journals-10018-1259

**Published:** 2018-05-01

**Authors:** Ruksana Raihan, Amirah Azzeri, Fatiha H Shabaruddin, Rosmawati Mohamed

**Affiliations:** 1 Department of Microbiology, Faculty of Medicine, AIMST University, Kedah, Malaysia; 2 Department of Social and Preventive Medicine Faculty of Medicine, University of Malaya, Kuala Lumpur, Malaysia; 3 Department of Pharmacy, Faculty of Medicine, University of Malaya Kuala Lumpur, Malaysia; 4 Department of Medicine, Faculty of Medicine, University of Malaya, Kuala Lumpur, Malaysia

**Keywords:** Cryptogenic, Hepatocellular carcinoma, Malaysia, Nonalcoholic fatty liver disease, Nonalcoholic steatohepatitis.

## Abstract

Hepatocellular carcinoma (HCC) is one of the leading causes of death globally. In Malaysia liver cancer is the eighth most common cause of cancer for both gender and fifth most common cause of cancer for males. Liver cancer is a cause of premature death in Malaysia: The trend from 1990 to 2010 was observed upward. Since 1990, the annual years of life lost (YLLs) from liver cancer have increased by 31.5%. Older persons are at higher risk and there is male predominance observed. Curative surgical resection, liver transplantation, and supportive symptomatic care, including percutaneous ethanol injection and radiofrequency ablation (RFA), and noncurative transarterial chemoembolization (TACE) are among available treatment facilities. Yet the survival rate is very poor as majority of patients present at very advanced stage. Hepatitis B virus (HBV) remained the leading cause of HCC in Malaysia. Several studies showed cryptogenic causes, which are mainly nonalcoholic fatty liver disease (NAFLD) and nonalcoholic steatohepatitis (NASH) among the predominant causes of HCC in Malaysia than hepatitis C virus (HCV), alcohol, or any other reason. This mainly correlates with the increasing incidence of diabetes and obesity in Malaysia.

**How to cite this article:** Raihan R, Azzeri A, Shabaruddin FH, Mohamed R. Hepatocellular Carcinoma in Malaysia and Its Changing Trend. Euroasian J Hepato-Gastroenterol 2018;8(1):54-56.

## INTRODUCTION

Hepatocellular carcinoma is the most common type of liver cancer arising from malignant hepatocytes. It is one of the leading causes of death globally. In 2012, HCC was the fifth most common cancer in men and ninth in women with 554,000 and 228,000 cases, respectively, according to the World Health Organization.^1^ It was estimated to cause nearly 745,000 deaths in 2012.^2^ In Malaysia, liver cancer is one of the ten most frequent cancers diagnosed.^3^ Malaysia is a multiethnic country with an estimated population of 31.56 million with 50.1% Malay, 22.6% Chinese, and 6.7% Indians.^4^ Average life expectancy is 78.5 years for females and 72.2 years for males. Primary liver cancer is the sixth most common cause of cancer and second leading cause of cancer-related death worldwide.^2^ Hepatocellular carcinoma is the most common form of liver cancer, accounting for 90% of the primary liver cancer worldwide. Hepatocellular carcinoma causes 670,000 deaths globally per annum, with up to 1,837 people every day or 76 people every hour. High prevalence of the main etiologies, HBV and HCV, are among the top three causes of cancer in Asia. Older persons are at higher risk and there is male predominance observed. Despite various clinical treatment options, such as potentially curative surgical resection, liver transplantation, supportive symptomatic care, including percutaneous ethanol injection and RFA, and noncurative TACE,^5^ the survival rate of HCC is very poor as the majority of patients present with advanced disease.^2^ Hepatocellular carcinoma is commonly diagnosed after the symptoms have presented or usually in intermediate or end stage of HCC.^6^ If early diagnosis was done and treatment was received, the survival rate could increase up to 50%. Management of advanced disease may include systemic therapy for palliation of symptoms,^5^ with one example of a systemic pharmacotherapeutic agent being sorafenib, a protein kinase inhibitor that inhibits tumor cell proliferation and angiogenesis.

## HEPATOCELLULAR CARCINOMA IN MALAYSIA

Liver cancer is the eighth most common cause of cancer for both gender and fifth most common cause of cancer for males in Malaysia. The annual mortality rate per 100,000 people from liver cancer globally is 11.4% and in Southeast Asia is 12.1%. In Malaysia, it is 6.1% in the year 2013, which showed an increase by 42.8% since 1990, an average of 1.9% a year.^7^ Liver cancer is a cause of premature death in Malaysia: The trend from 1990 to 2010 was observed upward. In the year 1990, the ranking of liver cancer as a cause of YLLs was 26, which became 22 in the year 2010.^8^ Since 1990, the annual YLLs from liver cancer have increased by 31.5%. Liver cancer disease burden in Malaysians measured by the YLLs per 100,000 people peaks at the age of 70 to 74 years for men and 75 to 79 years for women. It may affect boys as young as 10 to 14 years.^7^

There are generally scarce data describing the clinical management of patients with HCC in Malaysia within clinical practice. A descriptive cross-sectional analysis of a hospital-based case series on consecutive HCC patients who presented in 2013 at University Malaya Medical Centre (UMMC) was conducted. The UMMC is one of the teaching hospital and tertiary care referral centers for liver diseases in Malaysia.

There were 115 consecutive HCC patients presented at the hospital in 2013. The mean age (± standard error of mean) of patients at diagnosis with HCC is 61.14 (± 1.11) years, with 80% of patients being male (n = 92). One-third of the 115 patients were aged 61 to 70 years (n = 40, 34.8%). The majority of the patients are Chinese (n = 69, 60%), followed by Malay (n = 33, 28.7%) and Indian (n = 13, 11.3%). The most common etiology for HCC was HBV infection, which accounted for 51.3% of the patients, while 9.6% of patients had chronic HCV infection, which was comparable to a previous study.^9^

Patients were stratified into stages A, B C, and D based on the Barcelona Clinic Liver Cancer staging classification according to results of first diagnostic investigations comprising mainly five-phase computed tomography scan. However, due to variations in the availability of patients’ information from the hospital medical records, information on HCC stage at first presentation to UMMC was only available for 89 of the 115 patients.

At initial presentation to the hospital, 36/89 patients presented with stage D HCC. Out of 89 patients 20, 18, and 15 patients were diagnosed with stages B, C, and A respectively. All the 89 patients were further categorized according to the Child-Pugh score of cirrhosis. Of the 15 HCC patients who were in stage A during initial presentation, 13 of them had Child-Pugh A, while another 2 patients had Child-Pugh B score. Half of the patients (n = 10/20) in stage B HCC had Child-Pugh A score and another half (n = 10/20) had Child-Pugh B score of cirrhosis. In stage C, 5/18 and 13/18 HCC patients had Child-Pugh A and B score respectively. All patients (n = 36) in stage D had Child-Pugh C score at initial presentation to UMMC (Table 1).

**Table 1:** **Table 1:** Patients’ disease state at presentation to UMMC (n = 89)

*Characteristics*		*HCC patients*	
*Stage of HCC at diagnosis*			
A		15	
B		20	
C		18	
D		36	
*Stage of cirrhosis at presentation (Child-Pugh)*			
A		28	
B		25	
C		36	

Various health care resources were utilized in the clinical management of HCC patients. Resource use was categorized based on the health care setting of the treatment received, which comprised clinic visits, admission, RFA procedure, TACE procedure, and medical managements of liver cirrhosis related to HCC. The estimated direct medical costs for HCC were calculated from the healthcare provider’s perspective, using relevant unit costs as published in a previous study conducted in UMMC.^10^ The costs were inflation-adjusted according to a rate of 2.2%, to reflect the costs of HCC management in 2013. The estimated annual cost of managing all HCC patients (n = 115) at UMMC in 2013 is USD 886,995.

This analysis was based on retrospective data extracted from medical records. While there could be data gaps that may lead to incomplete description of the clinical treatment received, this analysis presents the best current information on the estimated costs of HCC in Malaysia. Future work should focus on refining these estimates to help inform future strategies for HCC in Malaysia.

## CHANGING TRENDS OF HCC IN MALAYSIA

Hepatitis B virus remained the major cause of HCC in Malaysia. Availability of effective HBV vaccine and nationwide vaccination programs, more affordable drugs, and a comprehensive national strategy will help to control and eliminate HBV in the near future. The increase in the number of HCC cases due to HBV is the reflection of already significant disease burden.^11^

Hepatitis C virus remains a concern for Malaysia as many infected persons are unaware of their infected status and due to the limited availability of effective treatment options. Hepatitis C virus disease burden in Malaysia is currently high and expected to rise steeply in coming decades.

Nonalcoholic fatty liver disease is increasing rapidly globally as well as in the Asia-Pacific region and is an emerging issue in Malaysia due to the rising incidence of obesity and diabetes, which are reaching epidemic proportions. Nonalcoholic steatohepatitis-induced HCC is another burning issue. The prevalence of NAFLD in the general population of Malaysia is 22.7%. Highest prevalence is among Indian (68.2%) and Malay (64.7%) males. A study by Goh et al^9^ conducted in University Malaya Medical Center (UMMC) from 2006 to 2009 on 348 HCC patients showed that Chinese people are most affected with HCC, which is around 68.7% followed by Malays 20.4% and Indians 10.9%. Male to female ratio was 3.4:1 and average age was 62.5 years. For Malay and Chinese patients, HBV remained the major cause but for Indians it was cryptogenic (29.0%) and alcohol (26.3%) induced mostly. Hepatitis C was more in Malays (18.3%) but less in Chinese and Indians. Overall etiology shows hepatitis B (59.2%) being the major cause followed by cryptogenic (16.4%) and hepatitis C (10.6%).^5^

A more recent cross-sectional study of all HCC (115) cases in 2015 in UMMC (data unpublished) also showed that predominant etiology was HBV (51.3%), cryptogenic (35.7%), HCV (9.6%), and alcohol (3.5%).

Another study by Norsa’adah and Nurhazalini-Zayani^11^ on 210 HCC patients from 1987 to 2008 showed age prevalence of >60 is maximum (38.1%). Hepatitis B virus (57.6%) remained the major cause of HCC followed by cryptogenic (28.1%) as second most common cause. At the time of diagnosis, most of the patients were symptomatic and presented at an advanced stage of HCC.^12^

All these above studies show that after HBV, cryptogenic/NAFLD/NASH is the predominant cause of HCC, which can be correlated with the increasing incidence of diabetes and obesity in Malaysia. The obesity statistics in the Asia-Pacific region showed that Malaysia is the most obese Asian country.^13^ Obesity prevalence was 43.1% in 2006, which became 60% in the year 2014. Prevalence of diabetes mellitus was 8.3% in 1996, 14.9% in 2006, and in 2011 became 20%.

## CONCLUSION

Hepatocellular carcinoma has long been a major public health problem with a very high mortality, especially in Asia. In Asia-Pacific region, HBV remains the major cause of HCC except Japan. A changing etiology of HCC in Asia and in Malaysia is largely due to the rising epidemic of obesity and diabetes being the underlying cause of NAFLD, especially its more aggressive form, NASH a major contributor to HCC. Prevention and control of viral hepatitis and curbing the growing epidemic of obesity and diabetes must be seen as a key part of HCC prevention. An optimal surveillance strategy also is crucial to detect early HCC and a proper insight on socioeconomic impact of HCC burden may help people to become aware of the situation.
